# Supplementary material for: In Vitro Measurement and Mathematical Modeling of Thermally-Induced Injury in Pancreatic Cancer Cells
Source: Cancers (Basel). 2023 Jan 21;15(3):655. doi: 10.3390/cancers15030655 (PMC9913239; doi:10.3390/cancers15030655)
Supplement: Supplementary file 1 [file cancers-15-00655-s001.zip › cancers-2125693-supplementary.pdf]

Supplementary Materials

We simulated thermal profiles within pancreatic tissue during microwave ablation with a 2.45 GHz water-cooled interstitial antenna using a coupled 3D electromagnetic-bioheat transfer model, as previously described[1]. The time-harmonic electromagnetic wave equation *(Equations S1)* was solved to determine the spatial electromagnetic profile in tissue, and the associated electromagnetic power absorbed in tissue is given by Equation S2. The Pennes' bioheat transfer equation *(Equation (S3))* was used to model spatial distribution of temperature at each time step.

$$\nabla^2\mathbf{E} + {\beta_0}^2(\varepsilon_r - \frac{j\sigma}{\omega\varepsilon_0})\mathbf{E} = 0 \qquad (S1)$$

$$Q_{mw}=\frac{1}{2}\sigma \mid \mathbf{E} \mid^2 \qquad (S2)$$

$$\rho c\frac{\partial T}{\partial t} = \nabla . k\nabla T + Q_{mw} - m_b c_b(T - T_b) \qquad (S3)$$

Where ρc represents volumetric heat capacity [J/m^3/K], k is thermal conductivity [W/m/K], T is the temperature in Kelvin, Q_{mw} is the time-averaged MW power absorbed in tissue [W/m^3], σ [S/m] is electrical conductivity, c_b is blood specific heat capacity [J/kg/K], T_b is the temperature of inflowing arterial blood [K], β_0 is the wavenumber in free space [1/m], ε_r is the relative permittivity, ε_0 is the permittivity of free space [F/m], and ω is the angular frequency [rad/s]. Following each time step of the transient solver, tissue properties were updated based on the current temperature, and the electric field and associated power loss density were re-computed, before solving the bioheat transfer equation for the next time step. The simulation proceeded in such an iterative manner for the 10 min of simulated MW ablation. Table S1 summarizes the nominal values of tissue physical properties (at 37 °C) used in our study. The temperature dependent thermal properties of pancreatic tissue have been reported in the literature, and these temperature dependencies were incorporated within our simulations. To our knowledge, the temperature dependent dielectric properties of pancreatic tissue across the ablative temperature range have not been reported; in this study, we applied a similar temperature dependency to pancreatic tissue dielectric properties as has been reported for other tissue types such as liver and lung[2], [3]. The model was implemented with COMSOL Multiphysics v6.0.

Table S1. Pancreas tissue biophysical properties employed in simulations

Tissue property	Nominal value at 37 °C	Reference for temperature dependency
Relative permittivity ε_r	57.2 [4]	Adapted from [2], [3]
Electrical conductivity σ	1.97 S/m [4]	Adapted from [2], [3]
Volumetric heat capacity ρc	3.73×10^6 J/m^3/K [5]	[5]
Thermal conductivity k	0.53 W/m/K [5]	[5]
Perfusion rate m_b	767 ml/min/kg [6]	Reduced to 0 above 60 °C to simulate microvascular stasis [7]

The initial temperature in all tissue domains was set to 37 °C. We used a first order scattering boundary condition at simulation boundaries and thermal insulation boundary condition around the periphery of the tissue domain. The metallic parts of MW applicator are highly conductive and thus perfect electric conductor boundary condition was applied to those surfaces. Additionally, a convective heat-flux boundary condition shown in *Equation S4* was applied to the exterior surface of MW applicator shaft to account for the cooling effects of circulating water through the MW applicator.

$$q_0 = h(T_{ext} - T) \qquad (S4)$$

Where heat transfer coefficient (h) and external temperature were selected to be 200 [W/ m^3/K] and 20 °C, respectively.

A non-uniform mesh with tetrahedral elements was employed in our study, with mesh density highest around the input port of MW applicator while being allowed to grow coarser around the applicator shaft. The maximal tetrahedral element edge length was 0.2 mm, 0.5 mm and 1–2 mm at MW input port, around the applicator shaft, and around tissue boundaries, respectively.

References

[1] H. Fallahi, D. Clausing, A. Shahzad, M. O'Halloran, M. C. Dennedy, and P. Prakash, "Microwave antennas for thermal ablation of benign adrenal adenomas," *Biomed. Phys. Eng. Express*, vol. 5, no. 2, p. 025044, Feb. 2019, doi: 10.1088/2057-1976/ab068b.

[2] J. Sebek, R. Bortel, and P. Prakash, "Broadband lung dielectric properties over the ablative temperature range: experimental measurements and parametric models," *Med Phys*, vol. 46, no. 10, pp. 4291–4303, Oct. 2019, doi: 10.1002/mp.13704.

[3] H. Fallahi, J. Sebek, and P. Prakash, "Broadband Dielectric Properties of Ex Vivo Bovine Liver Tissue Characterized at Ablative Temperatures," *IEEE Trans Biomed Eng*, vol. 68, no. 1, pp. 90–98, Jan. 2021, doi: 10.1109/TBME.2020.2996825.

[4] M. Matthaiou, S. Koulouridis, and S. Kotsopoulos, "A Novel Dual-Band Implantable Antenna for Pancreas Telemetry Sensor Applications," *Telecom*, vol. 3, no. 1, Art. no. 1, Mar. 2022, doi: 10.3390/telecom3010001.

[5] A. Mohammadi, L. Bianchi, S. Asadi, and P. Saccomandi, "Measurement of Ex Vivo Liver, Brain and Pancreas Thermal Properties as Function of Temperature," *Sensors*, vol. 21, no. 12, Art. no. 12, Jan. 2021, doi: 10.3390/s21124236.

[6] IT'IS Foundation, "Tissue Properties Database V4.0." IT'IS Foundation, 2018. doi: 10.13099/VIP21000-04-0.

[7] D. J. Schutt and D. Haemmerich, "Effects of variation in perfusion rates and of perfusion models in computational models of radio frequency tumor ablation," *Medical Physics*, vol. 35, no. 8, pp. 3462–3470, 2008, doi: 10.1118/1.2948388.
